# Methotrexate Induced Lung Injury in a Patient with Primary CNS Lymphoma: a Case Report

**DOI:** 10.4084/MJHID.2012.020

**Published:** 2012-04-02

**Authors:** Puneet Chhabra, Arjun Dutt Law, Vikas Suri, Pankaj Malhotra, Subhash Varma

**Affiliations:** Department of Internal Medicine, PGIMER Chandigarh 160012, India

## Abstract

Methotrexate is an antimetabolite commonly used in clinical practice for a variety of indications ranging from rheumatoid arthritis and other connective tissue disorders to high dose regimens in many malignancies. This folate antagonist has got a spectrum of toxicities among which gastrointestinal effects predominate. Lung injury is a well described but rare event and has been reported most often in patients who have been on long term oral therapy for rheumatic disorders. Acute lung injury in a patient receiving a high dose regimen for haematological malignancies has not been reported previously. We present one such case of methotrexate related acute lung injury in a patient of primary CNS lymphoma receiving high dose methotrexate.

## Introduction

Methotrexate has a broad spectrum of side effects ranging from gastrointestinal and haematological dysfunction to the less commonly described lung injury. The most commonly associated lung injury described in association with methotrexate use is pulmonary fibrosis that has been seen in patients who have been receiving long term oral therapy for rheumatoid arthritis. Less well known is the syndrome of acute lung injury that is seen in patients who are treated with high dose regimens as used in the management of osteosarcoma or haematological malignancies. Due to the already immunocompromised nature of these patients, it is important to clinically differentiate this potentially life threatening syndrome from other causes of acute lung injury such as bacterial, viral and fungal infections.

## Case Report

A 58- year- old female was admitted to a tertiary care centre in northern India for the treatment of primary CNS lymphoma. She was diagnosed on the basis of neuroimaging and a stereotactic brain biopsy which showed diffuse large B cell lymphoma. PET CT confirmed isolated CNS involvement. At admission she had an ECOG performance status of 4 because of the disease and neurosurgical intervention. Initially, CSF cytology was negative for malignant cells. Baseline high resolution CT chest was normal.

The patient was started on DeAngelis protocol which included five cycles of high dose intravenous methotrexate and vincristine, oral procarbazine and intrathecal methotrexate preceding whole brain irradiation and post radiation cytarabine. Due to her poor performance status, a lower dose of methotrexate (1gm/m^2^) was given in the first cycle. Four days after receiving the first cycle she developed fever and shortness of breath. Physical examination revealed presence of bibasilar crepts. Chest roentgenogram revealed bilateral lower zone infiltrates. Blood gases revealed hypoxia at room air. She was given injectable broad spectrum antibiotics and supplemental oxygen. All cultures (bacterial, fungal, viral) were sterile, β-d glucan was negative and the patient had a gradual improvement in symptoms over the next week. After the first cycle, CSF examination was repeated as per protocol and was positive for malignant cells. Accordingly, a decision was taken to give the usual dose of methotrexate (3.5 gm/m^2^) in the next cycle. A day after receiving methotrexate administration, the patient again developed breathlessness. Examination revealed diffuse bronchospasm and crepitations in both lung fields. She had no fever throughout this period. High resolution CT of the chest revealed bilateral patchy ground glass opacification as well as basal atelectasis.

Blood gases revealed severe hypoxemia, cultures were sterile, fungal and viral serologies were negative and patient had remained afebrile throughout this episode. A possibility of drug induced lung injury was kept. On the basis of clinical criteria devised by Searles and Mckendry the patient was diagnosed with methotrexate induced lung injury.^1^ She was managed with intravenous and inhaled corticosteroids in addition to inhaled bronchodilators. She improved with this management and was taken off oxygen after two weeks. Due to methotrexate toxicity further high dose chemotherapy was deferred and she was advised to proceed with radiation therapy. She was put on temozolomide therapy post whole brain radiotherapy. She remains on close follow- up one year later. Serial PET CT scans have shown remission of lymphoma and no recurrence after one year. No progression of lung injury or any fibrotic changes have been seen.

## Discussion

Methotrexate is an antimetabolite acting via folate inhibition. Therefore, it acts on tissues which have high rates of cellular division such as skin, gastrointestinal tract, liver and bone marrow. It is highly teratogenic and is classified as a category X drug in pregnancy. The effect of methotrexate on the lungs is less well studied. The spectrum of pulmonary toxicities involves hypersensitivity pneumonitis,^2^ interstitial fibrosis, pulmonary edema,^3^ bronchitis with airway hyperreactivity, bronchiolitis obliterans with organizing pneumonia (BOOP) and pulmonary nodules.^4^ The symptoms are nonspecific and include nonproductive cough, shortness of breath, chest pain and vague constitutional symptoms. The time of onset of symptoms after administration of methotrexate is highly variable. The injury does not appear to be dependent upon the dose. Cases have been reported where injury has occurred with doses as low as 7.5 mg to as high as 3600 mg. Even a single dose of methotrexate has been known to cause lung injury. The mechanism of lung injury has not been studied in detail however few studies have shown that cytokine modulation via P38-MAP kinase pathway may play an important role.^5^ It was also thought that polymorphism in the methylenetetrahydrofolate genes may be linked with methotrexate toxicity. Some studies show that polymorphism of the C677T methylenetetrahydrofolate gene is associated with increased toxicity of methotrexate while others do not.^6^,^7^ However a few more studies are required to prove the association between methylenetetrahydrofolate polymorphism and methotrexate toxicity. As most patients receiving methotrexate are also susceptible to infections due to their underlying disease, investigation for infectious causes of lung injury should be done in all patients. Bronchoalveolar lavage is important to rule out other infective etiologies however the findings in methotrexate induced lung injury are nonspecific and reveal a lymphocytic infiltrate. Radiographic findings reveal bilateral alveolar and interstitial infiltrate which are common in bibasilar regions but may be unremarkable.^8^,^9^,^10^ Lung biopsy reveals the presence of lymphocytes although occasional neutrophilic infiltrates may also be present.^11^ Eosinophilia, hyperplasia of Type 2 pneumocytes and interstitial fibrosis have also been described. Due to the absence of specific clinical, radiological and pathological findings, it is important to have a high index of suspicion and rule out other more common causes. Several criteria have been devised by Carson, Searle and Kremer (Table 1). Treatment involves withdrawal of methotrexate and administration of steroids. Spontaneous resolution despite continuation of the drug has also been described.^12^ Restarting methotrexate may lead to recurrence of lung injury although successful reintroduction of oral methotrexate has been described in two cases.^13^ Leucovorin rescue does not protect against methotrexate induced lung damage. Most patients have an uneventful recovery following lung injury,^14^ however, some may have persistent lung damage causing fibrosis and restrictive lung disease.

## Figures and Tables

**Figure 1:**
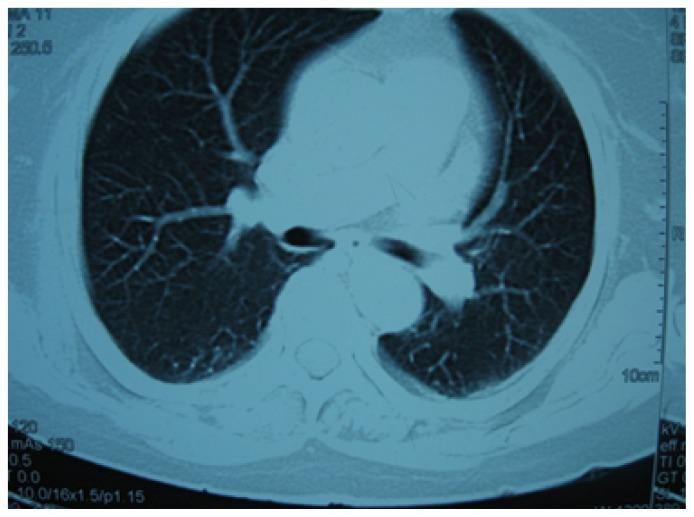
Figure showing normal tracheobronchial tree, vessels and normal lung parenchyma.

**Figure 2,3,4:**
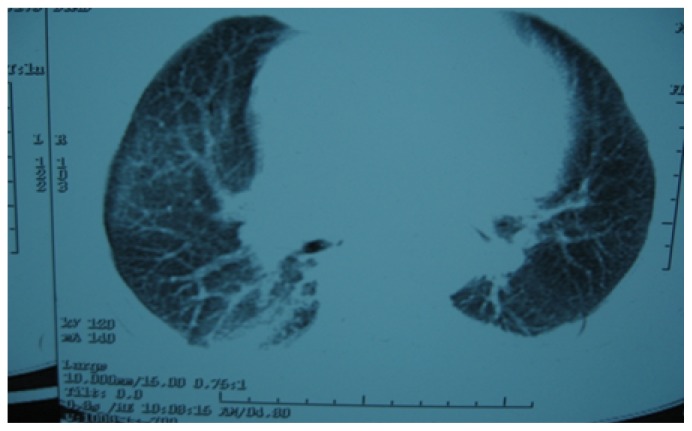

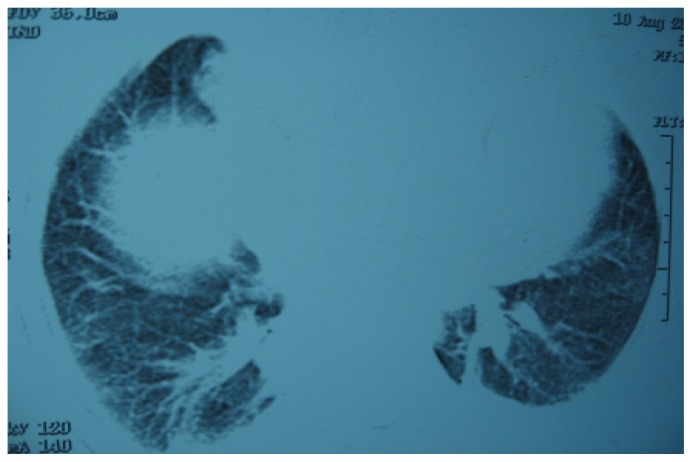

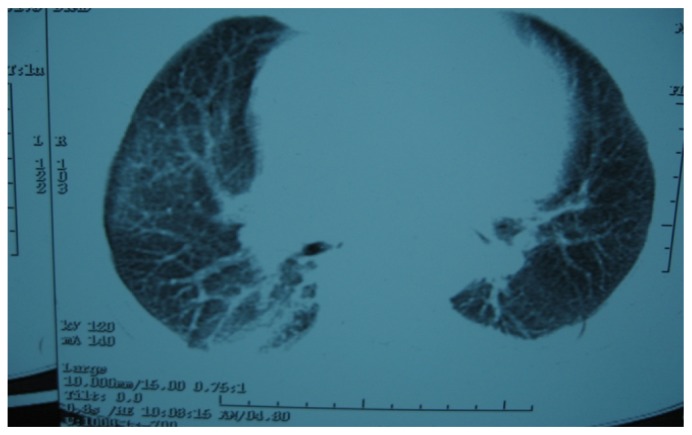
HRCT chest [after giving methotrexate], showing normal tracheobronchial tree, vessels but lung parenchymal changes in the form of b/l patchy ground glass opacities with b/l basal atelectasis.

**Table:** 

Carson et al. 1987	Searles et al. 1987	Kremer et al. 1997
Clinical 1. Clinical course consistent with hypersensitivity Radiology 2. Resolving infiltrates on chest roentgenogram(CXR) after discontinuing methotrexate Infection exclusion 3. Exclusion of infection or other pulmonary disease Histology 4. Pathology consistent with drug induced injury (i.e. hypersensitivity pneumonitis or toxic drug reaction) 3 or 4 out of the above 4 =**Probable MTX-P** 2 of above =**Possible MTX P** 1 of above= **Unlikely MTX-P**	Clinical 1. Acute onset of dyspnoea 2. Fever >38°c 3. Tachypnoea 28/min and non- productive cough Laboratory 4. WBC 15 × 10^9^/L (+/− eosinophilia) 5. PO_2_ on room air <55mm/Hg at admission Infection 6. Negative blood and sputum cultures (obligatory) Radiological 7. Pulmonary interstitial or alveolar infiltrates Pulmonary function tests 8. Restrictive pattern, decreased diffusion Histopathology 9. Bronchiolitis/interstitial pneumonitis with giant cells without evidence of pathogenic microorganisms 6 out of 9 criteria : **Definite MTX-P** 5 out of 9 criteria : **Probable MTX-P** 4 out of 9 criteria : **Possible MTX-P**	Major criteria Histology 1. Hypersensitivity pneumonitis by histopathological examination Radiology 2. Radiologic evidence of pulmonary interstitial or alveolar infiltrates Infection 3. Blood (if febrile) cultures ad initial sputum (if produced) cultures negative for pathogenic organisms^*^ Minor criteria Clinical 1. Shortness of breath<8 weeks 2. Dry cough Laboratory 3. O_2_ saturation <90% 4. WBC < 15, 000/mm^3^ Pulmonary fuction tests 5. DLCO 70% predicted ^*^microbiological criteria met if afebrile and no sputum production **Definite case of MTX-P:** Major criteria 1 Major criteria 2 and 3 and at least 3 minor criteria **Probable case of MTX-P:** Major criteria 2 and 3, and 2 minor criteria
